# Time in range, assessed with continuous glucose monitoring, is associated with brachial-ankle pulse wave velocity in type 2 diabetes: A retrospective single-center analysis

**DOI:** 10.3389/fendo.2022.1014568

**Published:** 2022-10-17

**Authors:** Hui Zhou, Wei Wang, Qiuyue Shen, Zhouqin Feng, Zhen Zhang, Haiyan Lei, Xinyi Yang, Jun Liu, Bin Lu, Jiaqing Shao, Ping Gu

**Affiliations:** ^1^ Department of Endocrinology, Jinling Hospital, Southern Medical University, Nanjing, China; ^2^ Department of Endocrinology, Jinling Hospital, School of Medicine, Nanjing University, Nanjing, China; ^3^ Department of Endocrinology, Jinling Hospital, Nanjing Medical University, Nanjing, China

**Keywords:** time in range, brachial-ankle pulse wave velocity, continuous glucose monitoring, type 2 diabetes, cardiovascular disease

## Abstract

**Aims:**

The aim of this retrospective single-center is to research the relationship between time in range(TIR), an important novel metric of glycemic control, assessed with continuous glucose monitoring(CGM) and brachial-ankle pulse wave velocity(BaPWV), a unique index of systemic arterial stiffness in type 2 diabetes.

**Methods:**

Study participants included 469 hospitalized patients with type 2 diabetes and no history of serious cardiovascular disease who underwent CGM and BaPWV measurements. TIR of 3.9-10.0 mmol/L was evaluated with CGM. BaPWV was measured by non-invasive arteriosclerosis detector and high baPWV was defined as a mean baPWV≧1800m/s. The spearman correlation and the partial correlation analysis were applied to analyze the correlation between TIR and baPWV. The binary logistic regression was used to examine the independent association of TIR and high BaPWV.

**Results:**

The presence of high baPWV was 32.2%. Compared with patients of low baPWV, those with high baPWV had significantly reduced TIR(P<0.001). With the increase of TIR tertiles, the prevalence of high BaPWV progressively decreased. Correlation analysis showed that TIR is inversely correlated with BaPWV. In a fully adjusted model controlling for traditional risk factor of CVD, TIR is associated with the presence of high BaPWV independent of HbA1c.

**Conclusion:**

TIR is correlated with BaPWV independent of HbA1c in patients with type 2 diabetes, confirming a link between TIR and arterial stiffness.

## 1 Background

The International Diabetes Federation (IDF) reported that 424.9 million people aged 20-99 years had diabetes in 2017, an increase of 281% over 2000 (1). In addition, the number of patients is expected to increase to 629 million by 2045. Compared with patients with normoglycemia, patients with type 2 diabetes have a substantially increased risk of developing cardiovascular diseases such as myocardial infarction or cerebral infarction (2–5). Cardiovascular events in patients with type 2 diabetes are the leading cause of the increased risk of early mortality and have become a growing threat to human health worldwide. Therefore, it is important to find strategies to prevent cardiovascular disease in patients with diabetes.

The pathological basis of CVD is arterial stiffness, including changes in arterial elasticity and abnormalities in arterial structure (6), and the changes in arterial elasticity occur earlier than arterial structure. When there is no corresponding symptom in the early stage of arterial stiffness, it is easy to be ignored. Therefore, detecting changes in arterial elasticity before structural changes in the arteries, i.e., before intimal thickening and plaque formation, is important for early clinical intervention and prognosis of arterial stiffness.

Brack-ankle pulse wave velocity (BaPWV) examination is a noninvasive test for arterial stiffness and has been shown to be an independent predictor of coronary artery disease and all-cause mortality in the general population (7). It is found that BaPWV is a sensitive indicator of early changes in arterial elasticity, which can screen for early arterial stiffness before the occurrence of arterial structural lesions (8). A higher baPWV indicates higher arterial stiffness (9).

HbA1c is the most widely used evaluation index of blood glucose control in clinical practice at present, reflecting the average level of blood glucose control in recent 2-3 months. There is evidence that HbA1c is closely related to vascular complications of diabetes mellitus. In a prospective trial of 3,642 patients, the UK prospective diabetes study (UKPDS) found that each 1% reduction in HbA1c was associated with a 37% reduction in the risk of diabetic microvascular complications, a 21% reduction in diabetes-related death, and a 14% reduction in myocardial infarction (10). However, some clinical trials have not shown that intensive glucose management based on HbA1c has a beneficial effect on the occurrence and development of CVD in diabetic patients (11–13). In fact, HbA1c has some limitations. The determination of HbA1c is affected by factors such as anemic, hemoglobinoopathy, pregnancy and race (14–16). In terms of accuracy, we found that HbA1c tends to be inconsistent with average blood glucose, often overestimating or underestimating blood glucose (17). In addition, HbA1c does not provide all the information, such as glycemic variability and hypoglycemia, both playing a crucial part in the development of CVD.

With the increasing application of continuous glucose monitoring(CGM) (18), we can obtain continuous, comprehensive and accurate glucose information. CGM can track blood glucose changes from day to week, understand hidden high/low blood glucose such as postprandial hyperglycemia, nocturnal hypoglycemia, dawn phenomenon and Somogyi phenomenon, which is difficult to be found by traditional blood glucose monitoring methods. More importantly, CGM covers a wide range of blood glucose information and can comprehensively reflect the characteristics of blood glucose fluctuations. CGM has derived many new indicators for the evaluation of blood glucose levels, among which time in range(TIR) has become a vane for the diagnosis and treatment of diabetes. TIR usually refers to the percentage of time within 24 hours that the glucose is in the range of 3.9 to 10.0 mmol/L, and it can reflect the blood glucose fluctuation state of patients all day more intuitively and vividly. TIR has also been proved to have highly consistent with HbA1c (19) and to compensate for the deficiency of HbA1c. Based on further research and clinical evidence, the American Diabetes Association (ADA) 2020 guidelines officially recommend TIR as a clinical endpoint and an emerging indicator for evaluating glycemic control (20). TIR has been shown to be significantly associated with diabetic microvascular complications, surrogate markers of macrovascular complications, all-cause and cardiovascular mortality (21–24).

However, the association between TIR and BaPWV has not yet been investigated. In this study, we enrolled 469 patients who underwent CGM and BaPWV measurements, and explored the relationship between TIR and BaPWV through statistical analysis.

## 2 Methods

### 2.1 Study population

We recruited 469 patients with type 2 diabetes who were hospitalized in the Department of Endocrinology, Jinling Hospital, Southern Medical University from October 2020 to July 2021. T2D was diagnosed according to 1999 World Health Organization (WHO) criteria. Exclusion criteria included diabetic ketoacidosis, hyperglycemic hyperotonic state, or recurrent severe hypoglycemic events within the previous 3 months, and a history of severe cardiovascular disease, malignancy, psychiatric illness, or severe liver or kidney dysfunction. All individuals underwent a 72-hour CGM and BaPWV test. The study was supported by the local ethics committee of Jinling Hospital.

### 2.2 Continuous glucose monitoring

All participants were monitored by CGM for 72 hours. A CGM(Mei QI, China) device was worn on the patient’s left upper arm or right arm and subcutaneous interstitial glucose was automatically recorded every 3 minutes (range:1.7-40mmol/L). The nurse intakes capillary blood glucose measurements more than four times a day to correct the instrument. The dynamic glucose parameters involved in our study were calculated by EasyGV Version9.0R2 published by The University of Oxford based on derived raw glucose data. TIR was defined as the percentage of time within the target glucose range (3.9-10.0mmol/L) within 24 hours. Daily glycemic variability (GV) parameters included M value, mean daily risk range (ADDR), standard deviation (SD) and coefficient of variation (CV).

### 2.3 Measurement of baPWV

Arteria stiffness was evaluated by measuring automatic BaPWV using a non-invasive vascular screening device(BP-203RPEIII,OMRON Medical Technology, Japan). The examination room was maintained as a stable temperature. The subjects were kept rest in supine position for at last 5-10 min in fasted condition, avoiding coffee or any exciting beverage or tobacco use before. Then a trained examiner placed pneumatic pressure cuffs on patient’s each ankle and each upper arm. The BaPWV was automatically calculated as the length of an arterial segment between the brochium and ankle(which was automatically calculated from the body height)divided by the transit time of the pulse wave. Based on the Japanese Circulation Society’s definition of arterial stiffness, we classified individuals with a BaPWV of 1800cm/s as having a higher level of arterial stiffness, who is considered a high-risk category and is known to increase the risk of cardiovascular and heart failure events. In addition to noninvasiveness, briefness, inexpensiveness, the BaPWV measurements have high reproducibility.

BaPWV was measured using an arterial stiffness monitoring device (BP-203RPEIII, OMRON Medical Technology, Japan). The examination room was maintained as a stable temperature. The subjects remained supine for 5-10 minutes and did not drink coffee or any exciting beverage or smoke before measurement. A trained examiner then places cuff bands around each ankle and upper arm of the patient. BaPWV is automatically calculated as the length of the artery segment between the brachial string and ankle (automatically calculated from body height) divided by the propagation time of the paralytic wave. According to the definition of arterial stiffness by the Japan Circulation Association (25), we classified those with a BaPWV of 1800cm/s as those with high degree of arterial stiffness. Therefore, we set BaPWV equal to 1800cm/s as the cut-off point of the grouping. In this study, patients were divided into two groups:BaPWV ≤ 1800cm/s was low arterial stiffness, and BaPWV > 1800cm/s was high arterial stiffness.

### 2.4 Clinical and biochemical information

We obtain physical examination data and biochemical indexes from electronic medical record system. Physical examination data included age, sex, course of diabetes, body mass index (BMI), systolic blood pressure (SBP), and diastolic blood pressure (DBP). Biochemical indicators included glycosylated hemoglobin (HbA1c), triglyceride (TG), total cholesterol (TC), high-density lipoprotein (HDL), low-density lipoprotein (LDL), albumin (Alb), urea nitrogen (BUN), creatinine (Scr), uric acid (UA). Blood samples from all participants were taken after an overnight fast. HbA1c was determined by high performance liquid chromatography (TOSOH HLC723G8 automatic HbA1C analyzer). We used a formula to calculate the estimated glomerular filtration rate (eGFR).

### 2.5 Statistical analysis

Statistical analysis was performed with SPSS25.0.Continuous variables conformed to normal distribution were shown as mean ± standard, while continuous variables with an abnormal distribution were expressed as median(upper and lower quartiles). Categorical data was expressed in count(percentage). The comparisons between groups were achieved by Student’s t-test, the Mann-Whitney test, the X²-test. The spearman correlation analysis was used to initially explore the relationship between TIR and BaPWV as a continuous variable, and then the partial correlation analysis was used to further explore the relationship between TIR and BaPWV after controlling for age, gender, diabetes duration, BMI and HbA1c(%). The binary logistic regression analysis was applied to the independent correlation between TIR and high BaPWV by adjusting age, sex, diabetes duration, SCr, BMI and HbA1c(%). Odds ratios(ORs) and 95% confidence intervals were listed. A P value of <0.05(two-tailed) was considered statistically significant.

## 3 Results

### 3.1 Clinical characteristics of the study objects

The baseline clinical characteristics of the 468 patients with type 2 diabetes were exhibited in **Table 1**. Mean age was 55 (47, 64) years, 67% were male, mean BMI was 24.9(22.7,27.3) kg/m², mean HbA1c level was 8.8(7.2,10.2)%, and estimated duration of type 2 diabetes was 7(2,14) years. The overall incidence rate of high BaPWV was prevalent in 32.2% of enrolled patients.

**Table 1:** The comparison of clinical characteristics by tertiles (T1-T3) of TIR.

	Tatal (n = 469)	T1 (n = 154)	T2 (n = 159)	T3 (n = 156)	X2/t/z	P值
**Age (years)**	55 (47,64)	56 (49,66)	55 (47.5,64.5)	54 (42,61)	13.72	**0.001**
**Male gender (%)**	314 (67%)	87 (55.8%)	107 (68.2%)	120 (76.9%)	15.929	**<0.001**
**Estimated duration of diabetes (years)**	7 (2,14)	10 (3,16)	8 (2.5,13.5)	4 (1,10)	17.163	**<0.001**
**Current smoker (%)**	131 (27.9%)	35 (22.4%)	44 (28%)	52 (33.3%)	4.603	0.1
**Body mass index (kg/m2)**	24.9 (22.7,27.3)	25.4 (23.2,27.73)	24.6 (22.05,26.65)	25.3 (22.5,27.8)	4.199	0.123
**Hypertension (n,%)**	51 (10.9%)	0	5 (3.2%)	46 (29.5%)	84.38	**<0.001**
**Systolic blood pressure (mmHg)**	130 (121,144)	133 (124,150)	130 (120,140)	130 (120.75,140)	10.793	**0.005**
**Diastolic blood pressure (mmHg)**	71.25 (78,87)	80 (73,87.75)	78 (70,86.5)	78 (71.75,89)	2.431	0.297
**Blood platelet**	201 (171,251)	215.5 (174,263.25)	198 (165.5,249.5)	200.5 (172,245.5)	0.395	0.821
**Alb (g/L)**	40.1 (37.8,42.6	39.6 (37.225,41.575)	40 (37.9,42.55)	40.8 (38.175,43.4)	9.765	**0.008**
**FBG (mmol/L)**	7.1 (5.7,8.9)	8.8 (7.2,10.9)	6.8 (5.75,8)	6.3 (5.2,7.3)	96.178	**<0.001**
**Total cholesterol (mmol/L)**	4.43 (3.71,5.12)	4.57 (3.82,5.33)	4.52 (3.74,5.06)	4.335 (3.595,5.13)	3.842	0.146
**LDL cholesterol (mmol/L)**	2.69 (2.07,3.32)	2.82 (2.08,3.42)	2.69 (2.11,3.31)	2.67 (2.04,3.32)	1.588	0.452
**HDL cholesterol (mmol/L)**	1.06 (0.9,1.25)	1.08 (0.90,1.31)	1.08 (0.91,1.25)	1.04 (0.9,1.19)	2.037	0.361
**Triglycerides (mmol/L)**	1.51 (1.04,2.30)	1.585 (1.023,2.53)	1.51 (1.055,2.19)	1.475 (1.028,2.26)	1.309	0.52
**Uric acid (umol/L)**	313.5 (258.25,390.5)	303.5 (234.25,368)	304 (259,378)	350.5 (282,410.25)	17.788	**<0.001**
**Estimated glomerular filtyation rate (mL)**	109.33 (96.90,121.38)	107.25 (97.2,118.78)	109.13 (97.67,122.45)	109.92 (93.55,125.62)	3.423	0.181
**HbA1c (%)**	8.8 (7.2,10.2)	9.7 (8.5,11.175)	8.7 (7.45,9.95)	7.6 (6.7,9.025)	3.451	<0.001
**Retinopathy (n, %)**	120 (25.6%)	37 (23.7%)	44 (28%)	24 (15.4%)	7.434	0.024
**Nephropathy DKD (n, %)**	71 (15.1%)	25 (16%)	29 (18.5%)	17 (10.9%)	3.637	0.162
**treatment (n,%)**						
**α-blockers**	2 (0.4%)	0	0	2 (1.3%)	4.03	0.133
**ACEI**	3 (0.6%)	0	0	3 (1.9%)	6.06	0.048
**ARB**	38 (8.1%)	0	2 (1.3%)	36 (23.1%)	70.57	**<0.001**
**β-blockers**	10 (2.1%)	0	3 (1.9%)	7 (4.5%)	7.58	0.023
**Calcium channel blockers**	41 (8.7%)	0	3 (1.9%)	38 (24.4%)	71.82	**<0.001**
**blockers**	16 (3.4%)	0	2 (1.3%)	14 (9.0%)	22.34	**<0.001**
**OHA**	359 (76.5%)	116 (74.4%)	115 (73.2%)	128 (82.1%)	4	0.135
**Insulin**	227 (48.4%)	69 (44.2%)	79 (50.3%)	79 (50.6%)	1.63	0.442
**FLP-CGM-derived metrics**						
**Mean (mmol/L)**	9.09 (7.97,10.65)	11.28 (10.62,12.48)	9.1 (8.64,9.64)	7.64 (7.02,8.2)	376.598	<0.001
**CV (%)**	24.74 (20.20,31.31)	24.71 (20.54,31.04)	28.59 (22.74,34.75)	20.92 (17.65,26.46)	48.464	<0.001
**SD**	2.36 (1.77,2.95)	2.78 (3.37,3.59)	2.63 (2.07,3.14)	1.54 (1.34,1.97)	178.893	<0.001
**MAGE (mmol/L)**	2.48 (1.88,3.21)	3.04 (2.42,3.87)	2.54 (1.92,3.31)	1.92 (1.53,2.44)	112.305	<0.001
**TIR 3.9-10 mmol/L**	68.62 (45.5,83.72)	38.25 (26.17,45.72)	68.03 (62.1,74.28)	88.27 (89.94,95.27)	416.001	<0.001
**TAR > 10 mmol/L**	22.44 (12.88,35.67)	43.4 (34.06,53.46)	25.02 (18.94,30.77)	9.61 (4.08,14.04)	332.994	<0.001
**TAR>13.9 mmol/L**	4.81 (0.14,13.80)	17.73 (10.7,28.53)	5.29 (1.9,9.27)	0 (0,1.43)	290.106	<0.001
**TBR<3.9 mmol/L**	0	0	0	0	11.628	0.003
**TBR<3.0 mmol/L**	0	0	0	0	8.475	0.014
**LBGI**	0.30 (0.27,1.08)	0.06 (0,0.49)	0.37 (0.13,1.31)	0.44 (0.15,1.13)	40.288	<0.001
**HBGI**	7.71 (4.39,11.62)	13.63 (11.19,17.71)	7.69 (6.13,9.21)	3.58 (2.17,4.46)	370.368	<0.001
**MODD (mmol/L)**	2.09 (1.51,2.88)	2.75 (2.07,3.73)	2.27 (1.73,2.99)	1.37 (1.07,1.82)	158.354	<0.001
**ADDR (mmol/L)**	22.99 (15.97,31.23)	32.49 (27.79,39.61)	23.73 (19.21,29.27)	12.87 (9.87,17.03)	274.589	<0.001

Continuous variables are presented as means ± SD and medians (lower and upper quartiles), and categorical variables are expressed as number (percentage).

UAE, urine microalbumin excretion; FPG, fasting plasma glucose; TC, total cholesterol; TG, triglyceride; BUN, blood urea nitrogen; Scr, serum creatinine; HbA1c, Hemoglobin A1C; BMI, body mass index; OHA, oral hypoglycemic agents; TIR, time in range; CV, coefficient of variation; SD, standard deviation; MAGE, mean amplitude of glycemic excursions; MODD, mean of daily differences; ADDR, average daily risk range. The bold type indicates statistical significance.

### 3.2 The comparison of clinical characteristics by tertiles of TIR

**Figure 1:**
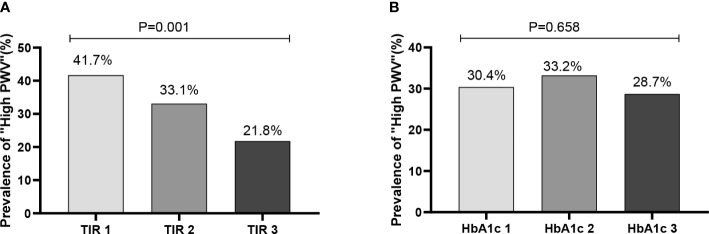
Prevalence of “high BaPWV” in different tertiles (T1-T3) of TIR and HbA1c. (**A**:TIR 1: TIR < 54.50%; TIR 2 :54.50% ≤ TIR≤79.67%; TIR 3: TIR > 79.67%; **B**: HbA1c 1: HbA1c < 7.2%; HbA1c 1 :7.2% ≤ HbA1c ≤ 10.2%; HbA1c 1: HbA1c > 10.2%).

Further analysis after dividing patients into groups was done according to tertiles of TIR (T1<54.50%;T2:54.50-79.67%;T3:>79.67%). The characteristics were shown in **Table 2**. Firstly, patients with the highest tertiles of TIR had lower FBG, HbA1c(%), SD, MAGE, LAGE, ADDR and M value(P<0.01). Of note, the prevalence of high arterial stiffness decreased with ascending tertiles of TIR (**Figure 1**). After grouping HbA1c tertiles (T1<7.2% (55mmol/mol);T2:7.2-10.2% (55 -88mmol/mol);T3:>10.2% (88mmol/mol)), there was no statistical significance in the prevalence of high arterial stiffness between groups (**Figure 1B**).

**Table 2:** The correlation of TIR and GV parameters with BaPWV by Spearman’s analysis.

	baPWV	
	r	P value
TIR	-0.244	<0.001
CV	0.110	0.017
SD	0.188	<0.001
MAGE	0.158	0.001
MODD	0.14	0.003
ADDR	0.205	<0.001
M value	0.228	<0.001

TIR, time in range; CV , coefficient of variation; SD, standard deviation; MAGE, mean amplitude of glycemic excursions; MODD, mean of daily differences; ADDR, average daily risk range.

### 3.3 The correlation of TIR and GV parameters with BaPWV

The correlation between TIR and BaPWV was performed with Spearman’s analysis. As shown in **Table 2**, the BaPWV was negatively correlated with TIR(r=-0.244, P<0.001) and was positively associated with MODD and SD. Furthermore, only the association between TIR and BaPWV maintained significant after adjusting for sex, age, diabetes duration, and BMI by the partial correlation analysis (r=-0.171, p<0.001) (**Table 3**).

**Table 3:** The correlation of TIR and GV parameters with BaPWV by partial correlation analysis.

	baPWV	
	r’	P value
TIR	-0.171	**<0.001**
CV	-0.04	0.406
SD	0.046	0.335
MAGE	0.023	0.624
MODD	0.045	0.354
ADDR	0.06	0.224
M value	0.056	0.242

TIR, time in range; CV , coefficient of variation; SD, standard deviation; MAGE, mean amplitude of glycemic excursions; MODD, mean of daily differences; ADDR, average daily risk range. The bold type indicates statistical significance.

### 3.4 Relationship between TIR and high BaPWV

Binary logistic regression analysis revealed that TIR was significantly associated with risk of high BaPWV (**Table 4**). The ORs for the risk of high BaPWV was 0.983(95%CI:0.975–0.991) in the crude model. After adding compounding variables including sex, age, diabetes duration, BMI, smoking, and hypertension (Model 1), the association between TIR and high BaPWV remained (OR = 0.985, 95%CI: 0.975-0.996, P = 0.005). We added Alb, UA,DR, DN, anti-hypertensive drugs and hypoglycemic drugs in the model 2 and model 3 of the binary logistic regression, the similar result was observed (P < 0.05). We added HbA1c in the model 4 of the binary logistic regression, the association between TIR and high BaPWV was observed (OR = 0.987, 95%CI: 0.975-0.999, P = 0.033) (**Table 4**).

**Table 4:** Association of TIR and BaPWV by binary logistic regression.

	Models	OR	95% CI		P value
TIR	Crude	0.983	0.975	0.991	<0.001
	Model 1	0.985	0.975	0.996	0.005
	Model 2	0.986	0.976	0.997	0.012
	Model 3	0.986	0.975	0.997	0.013
	Model 4	0.987	0.975	0.999	0.033

Crude was not adjusted;

Model 1 was adjusted for sex, age, diabetes duration, BMI, smoking, Hypertension;

Model 2 was adjusted for all variables in Model 1 plus Alb, UA,DR, DN;

Model 3 was adjusted for all variables in Model 2 plus anti-hypertensive drugs and hypoglycemic drugs

Model 4 was adjusted for all variables in Model 3 plus HbA1c.

## 4 Discussion

In the present study, we investigated the cross-sectional relationship between TIR and BaPWV in a relatively large population of individuals who underwent 72-h of CGM. Our study has several findings. we found a robust association between TIR and BaPWV independent of HbA1C. What’s more, the prevalence of high arterial stiffness decreased with ascending tertiles of TIR.

Cardiovascular disease is the main cause of morbidity and mortality in diabetic patients (26). Cardiovascular disease is estimated to be the largest component of health care spending for people with diabetes, accounting for 24% to 30% of hospital admissions and approximately one-third of deaths (27). So, the early detection and management of CVD is needed in order to combat the burden of disease complications.

Several studies have investigated the link between variability in glycemic levels and diabetes-related cardiovascular complications in recent years. In these studies, glycemic variability was assessed using fasting glucose or HbA1c over the follow-up period and showed that High levels of glycemic variability result in micro- and macrovascular complications and mortality regardless of their respective means glucose (28–30). With the increasing application of CGM, we can monitor patients’ dynamic blood glucose changes throughout the day and obtain new blood glucose management indicators. TIR is considered to be the most important indicator among CGM derived indicators. And the researchers found a correlation between TIR and HbA1c. By analyzing data from 18 randomized controlled trials (RCTS) involving patients with type 1 and type 2 diabetes, Vigersky et al. analyzed data from 18 randomized controlled trials (RCTs) involving patients with Type 1 and Type 2 diabetes and found a good correlation between TIR and HbA1c, found a good correlation between TIR and HbA1c, with 70% and 80% TIR equivalent to 6.7%(50mmol/mol) and 5.9%(41mmol/mol) of HbA1c value, respectively (19). In addition, researchers also explored the association between TIR and diabetes complications. Beck et al. found that each 10% increase in TIR was associated with a 64% and 40% reduction in the risk of retinopathy and albuminuria, respectively (31).

In the 2020 guidelines from the American Diabetes Association(ADA), TIR is advocated as a clinical endpoint and a metric for assessing glycemic control based on extensive clinical evidence (20). However, we lack direct evidence on the relationship between TIR and CVD. The long duration of cardiovascular events, the large number of samples required, and the high cost of CGM make it currently impossible to conduct such studies with a longitudinal design. In this context, early surrogate markers of CVD are particularly suitable for detecting TIR-CVD relationships. BaPWV, an arterial stiffness marker that reflects arterial elasticity, is a predictor of vascular injury severity and cardiovascular disease prognosis in patients with hypertension or diabetes. Furthermore, BaPWV is simple, noninvasive and suitable for routine clinical Settings. The Japanese Circulation Society proposed that the BaPWV of 1800 cm/s be used as the cut-off value for identifying subjects at high risk for CVD (32). Based on this cut-off value, approximately 33% of our study participants were defined as being at high risk for CVD. Correlation analysis revealed that the BaPWV was negatively correlated with TIR(r=-0.244, P<0.001) (**Table 1**). Logistic regression analysis revealed that TIR was inversely associated with the risk of CVD assessed by BaPWV (**Table 4**). After adjusting for sex, age, diabetes duration, Alb, BUN, EGFR and HbA1c, the relationship between TIR and BaPWV remained(P<0.05) (Model 3).

TIR was closely associated with arterial stiffness independent of HbA1c, and its molecular mechanism may be related to the effect of glucose fluctuations on oxidative stress. Previous studies have found that glucose variability is more likely than chronic persistent hyperglycemia to cause excessive production of inflammation and oxidative stress, leading to the formation of late AGE (33–35). As a result of AGE, collagen molecular crosslinking and collagen elasticity are reduced, leading to arterial stiffness (36). According to our research, CGM-derived metrics related to hyperglycemia such as TAR > 13.9 mmol/L and HBGI were significantly associated with high arterial stiffness. High postprandial glucose drift may be a causal factor for increased arterial stiffness. Therefore, focusing on reducing the magnitude of postprandial glucose drift based on CGM derived metrics may be important to reduce the risk of arterial stiffness and CVD development. A cross-sectional study found that recurrent hypoglycemia was related with preclinical atherosclerosis by measuring carotid and femoral artery ultrasound and flow-mediated arm dilation (37). In a case-control study, the effects of glycemic variability on endothelial function (as measured by flow-mediated dilation) and oxidative stress (based on plasma 3-nitrotyrosine and 8-iso-PGF2a urinary excretion rates) were studied in 27 patients with type 2 diabetes. It was found that although the stable group had higher mean glucose levels, the oscillations of glucose led to more severe endothelial dysfunction and more intense oxidative stress (36). At the genetic level, a study by Whist showed that transient hyperglycemia can lead to sustained epigenetic changes in the promoter region of NFKB, a pro-inflammatory gene, *in vitro* and in mice (38). While HbA1c only represents the average blood glucose level, these studies explain the reason why some clinical trials have failed to show a beneficial impact on the occurrence of CVD in diabetics when they adopted intensive glycemic management on the basis of HbA1c. Correspondingly, the disadvantage of glycosylated hemoglobin is that it cannot well reflect the fluctuation of blood glucose and the degree of hypoglycemia, while TIR can make up for the weakness of HbA1C in these aspects. In our study, the prevalence of arterial stiffness decreased significantly with the increase of TIR after grouping according to TIR, while there was no statistical significance in the prevalence between groups after grouping HbA1c (**Figure 1**).

This study has several limitations that should be noted. First, the study was a cross-sectional design and could not establish a causal relationship between TIR and BaPWV, although an opposite causal relationship seems unlikely. Second, although the models were adjusted for a mass of cardiovascular risk and lifestyle factors, residual confounding may still be present. Third, all subjects in this study underwent only 3 days of CGM, whereas previous studies have shown that increasing the duration of CGM monitoring improves the correlation of CGM data with glycemic index control. Thus, the results of our study should be interpreted with caution. Nevertheless, the preliminary data from this study may provide the basis for future longitudinal studies.

## 5 Conclusions

The significant association of TIR with BaPWV independent of HbA1c reveals the potential link between TIR and CVD, further supporting TIR as a valuable glucose metric and a reasonable outcome measure in clinical trials.

## Data availability statement

The original contributions presented in the study are included in the article/supplementary material. Further inquiries can be directed to the corresponding authors.

## Ethics statement

The studies involving human participants were reviewed and approved by the Ethics Committee of Jinling Hospital, Nanjing University. Written informed consent for participation was not required for this study in accordance with the national legislation and the institutional requirements.

## Author contributions

HZ, WW, and QS conceived and designed the research. HZ, ZF, ZZ, and HL analyzed and interpreted the data. HZ, XY, JL, and BL performed the statistical analysis. HZ wrote the manuscript. PG and JS critically revised the manuscript for key intellectual content. HZ, WW, and QS contributed equally to this paper. All authors contributed to the article and approved the submitted version.

## Funding

This work was supported by the National Natural Science Foundation of China(81873174), Key Research and Development Plan Project of Jiangsu Province - Social Development Projects(BE2020701).

## Acknowledgments

The authors thank all the involved clinicians, nurses, and technicians in the Department of Endocrinology, Jinling Hospital for dedicating their time and skill to the completion of this study.

## Conflict of interest

The authors declare that the research was conducted in the absence of any commercial or financial relationships that could be construed as a potential conflict of interest.

## Publisher’s note

All claims expressed in this article are solely those of the authors and do not necessarily represent those of their affiliated organizations, or those of the publisher, the editors and the reviewers. Any product that may be evaluated in this article, or claim that may be made by its manufacturer, is not guaranteed or endorsed by the publisher.

## Abbreviations

T2D, Type 2 diabetes; CVD, cardiovascular disease; CGM, Continuous glucose monitoring; BaPWV, Brachial-ankle pulse wave velocity; DR, Diabetic retinopathy; BMI, Body mass index; SBP, Systolic blood pressure; DBP, Diastolic blood pressure; BUN, Blood urea nitrogen; Scr, Serum creatinine; UA, Uric acid; TC, Total cholesterol; TG, Triglyceride; HDL, High-density lipoprotein; LDL, Low-density lipoprotein; Alb, Serum albumin; HbA1c, Hemoglobin A1c; MDSI, Multiple daily subcutaneous injection; TBR, Time below range; TIR, Time in range; TAR, Time above range; GV, Glucose variability; ADDR, Average daily risk range; MAGE, Amplitude of glucose excursions; SD, Standard deviation; CV, Coefficient of variation; AUC, Area under the curve.
